# Supplementary material for: MetaRibo-Seq measures translation in microbiomes
Source: Nat Commun. 2020 Jun 29;11:3268. doi: 10.1038/s41467-020-17081-z (PMC7324362; doi:10.1038/s41467-020-17081-z)
Supplement: Supplementary file 10 — Supplementary Data 7 [file 41467_2020_17081_MOESM10_ESM.zip › File2/Confidence_VeryHigh_Taxonomy/321344_out.krona.html]

Javascript must be enabled to view this page.

members
magnitude
magnitudeUnassigned
count
unassigned
taxon
rank

321344\_out

4

2
4
superkingdom

32066
phylum
1

203490
1
class

203491
order
1

1129771
1
family

32067
1
genus

712368
species

SRS019696\_contig\_number\_5921
1

phylum
2
1239

class
1
186801

186802
order
1

186803
1
family

SRS147377\_contig\_number\_contig-100\_8215.49668

909932
1
class

1
order
1843489

1
family
31977

29465
genus
1

species

SRS020334\_contig\_number\_contig-100\_15729.15729
1
39778

976
phylum
1

class
1
200643

order
1
171549

171550
family
1

239759
1
genus

1896970
species

SRS014235\_contig\_number\_contig-100\_14047.200058
1
